# Dietary intake of luteolin is negatively associated with all-cause and cardiovascular mortality in chronic kidney disease patients

**DOI:** 10.1186/s12889-024-19458-x

**Published:** 2024-07-30

**Authors:** Xiaotian Yao, Zhengxi Zhou

**Affiliations:** 1 The Division of Nephrology, The Sixth Affiliated Hospital, Sun Yat-Sen University, Guangzhou, Guangdong China; 2 Biomedical Innovation Center, The Sixth Affiliated Hospital, Sun Yat-sen University, Guangzhou, Guangdong China; 3 Department of Urology, Ningbo Mingzhou Hospital, Ningbo, Zhejiang China

**Keywords:** Luteolin, CKD, NHANES, All-cause mortality, Cardiac mortality

## Abstract

**Background:**

Luteolin (Lut), a flavonoid present in the daily diet, exhibits potent anti-inflammatory and renoprotective effects. However, the association between Lut and chronic kidney disease (CKD) remains uncertain. The objective of this study is to explore the potential correlation.

**Methods:**

A total of 2,393 CKD patients were enrolled in a prospective cohort in the National Health and Nutrition Examination Survey (NHANES). A 24-h dietary recall was utilized to estimate the intake of dietary Lut based on the type and amount of food consumed. The National Death Index mortality data was utilized to ascertain all-cause and cardiac mortality (as of December 27, 2023). Cox proportional hazards model was used to estimate the relationship between Lut intake and mortality risk.

**Results:**

The median Lut intake was 0.305 mg/day, with interquartile range was 0.105–0.775 mg/day. During the follow-up period (median, 93 months), 682 all-cause deaths (217 cardiovascular disease [CVD] deaths) were recorded. Per unit increase in Lut intake reduced all-cause mortality by 27% (*P* < 0.001) and cardiac mortality by 34% (*P* = 0.01) in CKD patients. There was an inverse dose–response association between Lut intake (range: 0–8.945 mg/day) and mortality risk. Consistent results were also shown when stratified by age, sex, race, marital status, body mass index, CKD stage, urine protein creatinine ratio strata, CKD progression risk, hypertension, and CVD.

**Conclusion:**

Dietary Lut intake is associated with a reduction in all-cause and cardiac mortality among CKD patients, potentially attributable to the anti-inflammatory characteristics of Lut.

## Introduction

Chronic kidney disease (CKD) is a significant contributor to morbidity and mortality from non-communicable diseases, both as a global public health concern and as a huge medical and financial burden [1]. CKD patients have significantly higher cardiovascular morbidity and mortality [2]. In patients with advanced CKD (stage 4) as well as end-stage kidney disease (stage 5), cardiovascular mortality accounts for approximately 40% to 50% of all deaths [3]. CKD induces a persistent, systemic inflammatory condition and subsequently triggers a remodeling process in the blood vessels and heart. This remodeling process contains atherosclerotic lesions, vascular calcification, vascular senescence, myocardial fibrosis, and cardiac valve calcification [3]. CKD, as an inflammatory disease, is closely related to the daily diet. Therefore, a healthy dietary pattern can slow the CKD progression and avoid premature cardiovascular complications and death.

Luteolin (Lut) is a flavonoid found in the daily diet [4], and their main sources include tea (black and green tea), vegetables (onions, celery, etc.), fruits (apples, grapes, etc.) and wine [5]. Previous studies have shown that Lut has benefits, including cardiovascular protection [6], improved insulin sensitivity [7], and anti-inflammatory effects [8]. In diabetic animal models, Lut increases insulin sensitivity [9] and alleviates the deterioration of renal function [10]. Lut exhibits therapeutic effects in a number of animal models of heart disease, such as improving heart function in heart failure [11], reversing atherosclerosis in coronary artery disease [12], and encouraging cell protection in myocardial ischemia/reperfusion (I/R) injury [13]. In general, research has shown Lut's protective effects on the renal and cardiovascular system. However, few population-based research studies have looked into its connection to similar clinical outcomes.

The purpose of this study was to use data from the National Health and Nutrition Examination Survey (NHANES) to examine the association between dietary Lut intake and cardiovascular and all-cause mortality in CKD patients. First, we used restricted cubic splines (RCS) to investigate the association between dietary Lut intake and cardiovascular disease (CVD) and all-cause mortality in patients with CKD. Then, the individual effects of Lut were analyzed using weighted COX regression models and sensitivity analyses. Finally, survival analysis was utilized to explore the relationship between Lut intake and survival in CKD patients.

## Materials and methods

### Study population

Data from the NHANES conducted from 2007 to 2010 and 2017 to 2018 were employed in the study. NHANES is a comprehensive survey that collects data from a diverse sample of the American population through a complex and systematic sampling technique, ensuring national representation. The study plan received approval from the ethical review board of the National Center for Health Statistics (NCHS), which was responsible for overseeing the survey's administration. Written informed permission was acquired from each participant. All the analyses used weighted samples ("wtmec2yr") and considered the stratification and clustering of the design, so as to represent an estimation of the population in the United States.

**Fig. 1:**
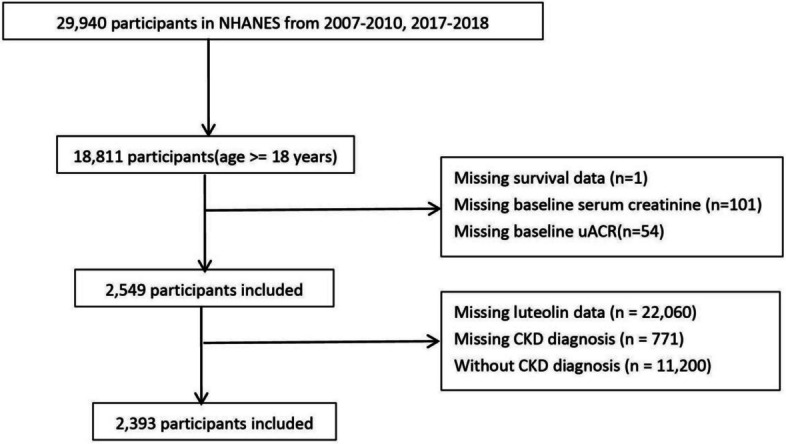
Flowchart of study population

This study includeindividuals aged 18 years or above, selected from three NHANES cycles (2007–2008, 2009–2010 and 2017–2018). Subsequently, participants meeting the following criteria were excluded: (1) those with missing data regarding Lut data (*n* = 6,238); and (2) those with missing data regarding CKD diagnosis (*n* = 771) or without CKD (*n* = 11,200); and (3) those with missing data regarding survival data (*n* = 1); and (4) those without baseline serum creatinine (*n* = 101); and (5) those without baseline urine albumin-to-creatinine ratio (uACR) (*n* = 54). As a result, the analysis included a total of 2,393 CKD patients (Fig. 1).

### Definition of CKD

CKD is characterized by an estimated glomerular filtration rate (eGFR) below 60 mL/min/1.73 m^2^ for a period exceeding 3 months, irrespective of other causes [14]. Moreover, the identification of albuminuria, characterized as a uACR exceeding 30 mg/g in two out of three spot urine samples [14], can serve as an indicator for kidney impairment in different kidney disorders. To calculate the eGFR, we can utilize the equation provided by the 2009 Chronic Kidney Disease Epidemiology Collaboration (CKD-EPI) [15].

### Dietary Lut intake assessment

In the study, the mean value of Lut data from the NHANES for two days was utilized. The first day Lut intake recall interview took place at the Mobile Inspection Center (MEC), and the second interview was conducted via telephone within a period of 3–10 days. The Lut was calculated by the United States Department of Agriculture (USDA) Food and Nutrient Database for Dietary Studies (FNDDS) for NHANES. Lut data are derived from the average value of two days.

### Covariates

A family interview questionnaire provided the demographic information about the study group. The participants were categorized into two groups based on their age: over 60 years, and under 60 years. The ethnicity was classified into five categories: White, Black, Mexican American, and other race. We categorized daily family income into three groups based on the poverty ratio: > 3.5 (considered as high income), 1.3–3.5 (considered as middle income), and < 1.3 (considered as low income). Marital status was divided into two categories: coupled (married or in a committed relationship) and single (not married or in a committed relationship). There were four categories used to classify education levels: college graduate or higher, some college or associates degree, high school graduate, and less than high school graduate. We categorized body mass index (BMI) into two groups: ≥ 30 (considered as obesity), < 30 (considered as non-obese). Drink status was categorized into two groups: drinking and not drinking. Smoking status was categorized into three groups: former, current and never. During the physical examination, essential biometric data like blood pressure (BP), stature, and BMI were collected. The calculation of BMI involves dividing weight in kilograms by the square of height in meters. We also included laboratory data, such as serum creatinine, serum uric acid, serum urea, serum carbon dioxide, uACR, etc.

### Outcome assessment

The survival data were monitored and recorded until the conclusion of the observation period on December 27, 2023. The follow-up time is defined as several months from family interview to death or end of follow-up. The National Death Index (NDI) was used to determine the mortality status during follow-up. All-cause death, CVD and other causes of death were determined by the International Classification of Diseases (ICD) 10.

### Statistical analysis

To ensure that the estimates are representative at a national level, this study considered the complex sampling design of the NHANES, taking into account sample weighting, clustering, and stratification in all analyses. The baseline characteristics of CKD patients were statistically described. Weighted medians (IQR) were used to present continuous variables, along with their corresponding CIs. Weighted percentages were used to present categorical variables. Comparisons between groups were performed using multiple sample test for continuous variables, and the Chi-squared test or Fisher's exact test for categorical variables.

The issue of missing data was addressed using a specialized multilevel approach tailored for survey data, employing the R package "jomo" [16]. To generate 10 imputed data sets, a Gibbs sampling procedure was utilized, preceded by a burn-in period of 500 iterations and followed by 100 updates. This process ensured that the imputed data sets were stochastically independent from each other. A complete case approach was utilized for statistical analysis.

RCS regression was used to investigate potential non-linear relationships between dietary intake of Lut and the risk of mortality in CKD patients. We evaluated the existence of non-linearity by conducting the likelihood ratio test. Survey-weighted univariable and multivariable cox regression analyses were carried out to further investigate the relationship between dietary intake of Lut and the risk of mortality in CKD patients. Potential confounders were taken into account in these analyses, including age, gender, marital, poverty, hypertension, HbA1c, history of CVD, serum creatinine, and uACR.

In order to examine specific groups that might be more susceptible to disparities related to demographics, a stratified analysis was performed, taking into account factors such as age, serum creatinine, uACR, BMI, uric acid, blood urea nitrogen, bicarbonate, hemoglobin (Hb), albumin, and HbA1c. A *p*-value was calculated and presented to show the importance of the interaction between each stratification factor.

R version 4.3.1 (R Foundation for Statistical Computing, Vienna, Austria) was utilized for the statistical analyses. Statistical significance was determined by considering a *p*-value of less than 0.05 in all two-tailed tests.

## Results

This study included 2,393 CKD patients aged 18 years or older. Baseline characteristics of the study population are summarized by Lut intake tertiles, with weighted mean age 60.8 years (95%CI 60–61.8 years) and 1121 males (42.1%, 95%CI 38–46.3%). The median Lut intake was 0.305 mg/day, with an interquartile range was 0.105–0.775 mg/day. Participants with higher Lut intake were more likely to be college graduates or above, couple, white, never smoked or drunk, HbA1c < 7, without CVD history, moderate risk of CKD prognosis, a higher energy intake and a higher income. No significant difference among the groups was seen for serum creatinine, uric protein, eGFR, uric acid, blood urea nitrogen, obesity, or higher serum albumin (Table 1).

**Table 1:** Baseline Characteristics of the study population

Characteristic	Total	Lut intake (mg/day)			*P-*value
		Tertile 1	Tertile 2	Tertile 3	
		[0–0.16)	[0.16–0.574)	[0.574–8.945]	
Age	60.89(59.99,61.79)	60.32(58.77,61.86)	61.47(59.55,63.40)	60.83(59.15,62.52)	0.7
Age strata					0.73
< 60	39.32(34.76,43.89)	39.36(33.70,45.02)	37.74(32.58,42.90)	40.70(35.61,45.78)	
> = 60	60.68(55.35,66.01)	60.64(54.98,66.30)	62.26(57.10,67.42)	59.30(54.22,64.39)	
Gender					0.17
Male	42.11(37.95,46.27)	44.74(39.88,49.59)	38.54(34.99,42.10)	43.15(38.13,48.17)	
Female	57.89(52.69,63.09)	55.26(50.41,60.12)	61.46(57.90,65.01)	56.85(51.83,61.87)	
BMI, kg.m2	30.47(30.04,30.91)	30.59(29.96,31.23)	30.17(29.58,30.76)	30.65(29.80,31.50)	0.58
SBP, mmHg	130.86(129.60,132.11)	132.51(130.89,134.13)	129.72(127.51,131.93)	130.53(128.00,133.06)	0.16
DBP, mmHg	69.31(68.37,70.25)	69.21(67.98,70.43)	68.33(66.98,69.68)	70.27(68.69,71.86)	0.13
energy intake, kcal/day	1861.08(1815.94,1906.21)	1792.35(1699.81,1884.89)	1838.46(1777.63,1899.30)	1936.58(1870.00,2003.16)	0.05
					< 0.001
< 1500	33.49(30.02,36.96)	41.34(36.00,46.68)	33.40(29.56,37.24)	27.24(22.85,31.62)	
> = 1500	66.51(60.43,72.58)	58.66(53.32,64.00)	66.60(62.76,70.44)	72.76(68.38,77.15)	
Hypertension					0.29
hypertension	67.98(61.26,74.70)	70.85(66.92,74.79)	64.86(58.95,70.78)	68.43(63.02,73.84)	
normal	32.02(28.55,35.49)	29.15(25.21,33.08)	35.14(29.22,41.05)	31.57(26.16,36.98)	
Diabetes					0.32
Diabetes	36.01(32.31,39.71)	41.06(37.20,44.93)	33.92(29.97,37.87)	33.77(28.74,38.80)	
IGT	3.22( 2.41, 4.03)	2.80(1.70,3.90)	3.78(2.21,5.35)	3.07(1.57,4.57)	
IFG	5.28( 3.96, 6.60)	5.12(2.61,7.64)	5.61(3.07,8.16)	5.10(3.24,6.97)	
no	55.49(50.39,60.60)	51.01(46.91,55.12)	56.69(52.57,60.80)	58.05(53.45,62.66)	
CVD history					0.02
no	76.83(70.44,83.23)	72.73(68.95,76.50)	77.89(74.52,81.25)	79.22(76.10,82.33)	
yes	23.17(20.38,25.95)	27.27(23.50,31.05)	22.11(18.75,25.48)	20.78(17.67,23.90)	
Education					< 0.0001
High school graduate	28.79(24.45,33.13)	31.87(27.45,36.29)	28.17(22.89,33.45)	26.86(22.61,31.11)	
< High school graduate	21.06(18.39,23.73)	30.77(26.75,34.79)	19.86(16.36,23.36)	14.28(10.72,17.85)	
Some college or associates degree	28.56(25.16,31.96)	25.59(20.88,30.30)	27.46(23.27,31.64)	31.93(27.11,36.75)	
College graduate or above	21.59(18.34,24.84)	11.77( 8.91,14.62)	24.51(19.38,29.65)	26.93(21.74,32.11)	
Ethnicity					< 0.001
White	72.10(63.49,80.72)	68.34(63.16,73.52)	72.80(68.00,77.61)	74.52(69.91,79.14)	
Other	9.32( 7.88,10.77)	10.70(7.81,13.60)	7.39(5.16, 9.62)	9.93(6.76,13.09)	
Mexican American	7.27( 5.47, 9.07)	5.48(3.56, 7.41)	7.57(4.88,10.26)	8.45(6.07,10.82)	
Black	11.30( 9.33,13.28)	15.47(12.29,18.65)	12.24( 9.37,15.11)	7.11( 5.18, 9.04)	
Marital					0.02
couple	59.38(53.00,65.76)	53.42(49.01,57.84)	59.89(54.31,65.48)	63.73(58.43,69.04)	
single	40.62(36.69,44.55)	46.58(42.16,50.99)	40.11(34.52,45.69)	36.27(30.96,41.57)	
Poverty					< 0.0001
1.3–3.5	43.41(39.06,47.76)	49.64(44.99,54.28)	40.72(36.88,44.55)	40.77(37.00,44.53)	
< 1.3	21.63(19.26,24.00)	29.14(25.88,32.40)	20.93(17.33,24.52)	16.19(13.33,19.05)	
> 3.5	34.96(30.86,39.06)	21.22(17.05,25.40)	38.36(33.46,43.25)	43.04(38.22,47.86)	
Obesity					0.88
no	51.41(46.87,55.96)	51.31(46.49,56.14)	52.40(47.51,57.28)	50.63(45.26,55.99)	
yes	48.59(43.43,53.74)	48.69(43.86,53.51)	47.60(42.72,52.49)	49.37(44.01,54.74)	
Smoking					< 0.0001
former	32.92(29.56,36.27)	31.23(26.86,35.61)	32.12(28.22,36.03)	34.98(31.01,38.94)	
now	14.80(12.66,16.94)	23.34(20.11,26.58)	12.78(10.65,14.92)	9.70( 7.19,12.20)	
never	52.28(47.31,57.26)	45.43(40.20,50.65)	55.10(50.28,59.92)	55.33(50.60,60.06)	
Drinking					< 0.0001
no	44.54(40.09,48.99)	53.26(47.96,58.56)	45.96(40.57,51.35)	36.24(31.61,40.87)	
yes	55.46(49.34,61.57)	46.74(41.44,52.04)	54.04(48.65,59.43)	63.76(59.13,68.39)	
Serum creatinine, umol/L	97.63(94.53,100.72)	98.93(94.76,103.11)	97.66(93.18,102.13)	96.54(90.71,102.38)	0.8
eGFR					0.39
< = G3a	85.43(78.43,92.42)	83.55(80.37,86.73)	85.53(82.71,88.35)	86.85(83.74,89.95)	
> = G3b	14.57(12.93,16.22)	16.45(13.27,19.63)	14.47(11.65,17.29)	13.15(10.05,16.26)	
Serum uric acid, umol/L	354.84(349.50,360.19)	357.86(351.90,363.82)	352.39(343.75,361.03)	354.58(344.48,364.68)	0.48
Blood urea nitrogen, mmol/L	6.49(6.34,6.64)	6.40(6.20,6.60)	6.43(6.18,6.69)	6.62(6.31,6.93)	0.51
Serum bicarbonate, mmol/L	25.32(25.12,25.52)	25.21(24.93,25.50)	25.24(24.96,25.51)	25.48(25.16,25.80)	0.33
Serum triglycerides, mmol/L	1.97(1.87,2.08)	2.01(1.88,2.15)	1.94(1.76,2.13)	1.96(1.82,2.10)	0.77
Serum fast total cholesterol, mmol/L	4.95(4.88,5.02)	4.92(4.82,5.02)	4.91(4.81,5.01)	5.02(4.90,5.14)	0.24
Serum HDL cholesterol, mmol/L	1.35(1.32,1.37)	1.32(1.28,1.36)	1.36(1.31,1.41)	1.36(1.32,1.40)	0.29
Hb, g/dL	13.87(13.75,14.00)	13.78(13.55,14.00)	13.78(13.63,13.94)	14.03(13.86,14.21)	0.07
Albumin, g/L	40.98(40.76,41.21)	40.61(40.16,41.05)	41.07(40.80,41.34)	41.21(40.91,41.52)	0.05
HbA1c	6.16(6.09,6.23)	6.27(6.15,6.40)	6.15(6.02,6.27)	6.08(5.97,6.20)	0.14
HbA1c strata					0.03
HbA1c < 7	84.04(76.78,91.29)	79.62(75.40,83.84)	85.73(82.62,88.85)	86.10(82.56,89.64)	
HbA1c > = 7	15.96(13.63,18.30)	20.38(16.16,24.60)	14.27(11.15,17.38)	13.90(10.36,17.44)	
uACR, mg/g	41.25(12.13,96.47)	45.11(17.53,110.00)	39.31(11.15, 80.46)	39.18(11.20, 96.98)	0.09
CKD ACR					0.1
A1	34.66(29.67,39.65)	28.97(24.08,33.86)	38.03(31.08,44.99)	36.26(31.53,40.99)	
A2	55.25(50.41,60.08)	60.18(55.54,64.81)	53.30(46.44,60.17)	52.99(47.86,58.12)	
A3	10.09( 8.53,11.66)	10.85(7.86,13.84)	8.66(6.74,10.59)	10.75(8.16,13.34)	
CKD prognosis					0.01
moderate risk	72.39(66.29,78.48)	69.02(65.96,72.09)	74.47(71.38,77.56)	73.26(69.78,76.75)	
high risk	18.05(15.70,20.41)	18.66(16.08,21.24)	15.52(13.01,18.03)	19.81(15.98,23.63)	
very high risk	9.56( 8.32,10.80)	12.32(10.06,14.58)	10.01( 7.61,12.42)	6.93( 4.69, 9.17)	
Follow-up time, month	78.41(74.48,82.34)	71.42(65.79,77.05)	85.38(78.91,91.84)	77.88(73.26,82.51)	0.003

Data were presented as weighted percentages or means (95% confidence intervals)

*Abbreviation*: *SBP *systolic blood pressure, *DBP* diastolic blood pressure, *BMI* body mass index, *Hb* hemoglobin, *uACR* urine albumin creatine ratio, *eGFR* estimated glomerular filtration rate, *IGT* impaired glucose tolerance, *IFG* impaired fasting glucose

**Fig. 2:**
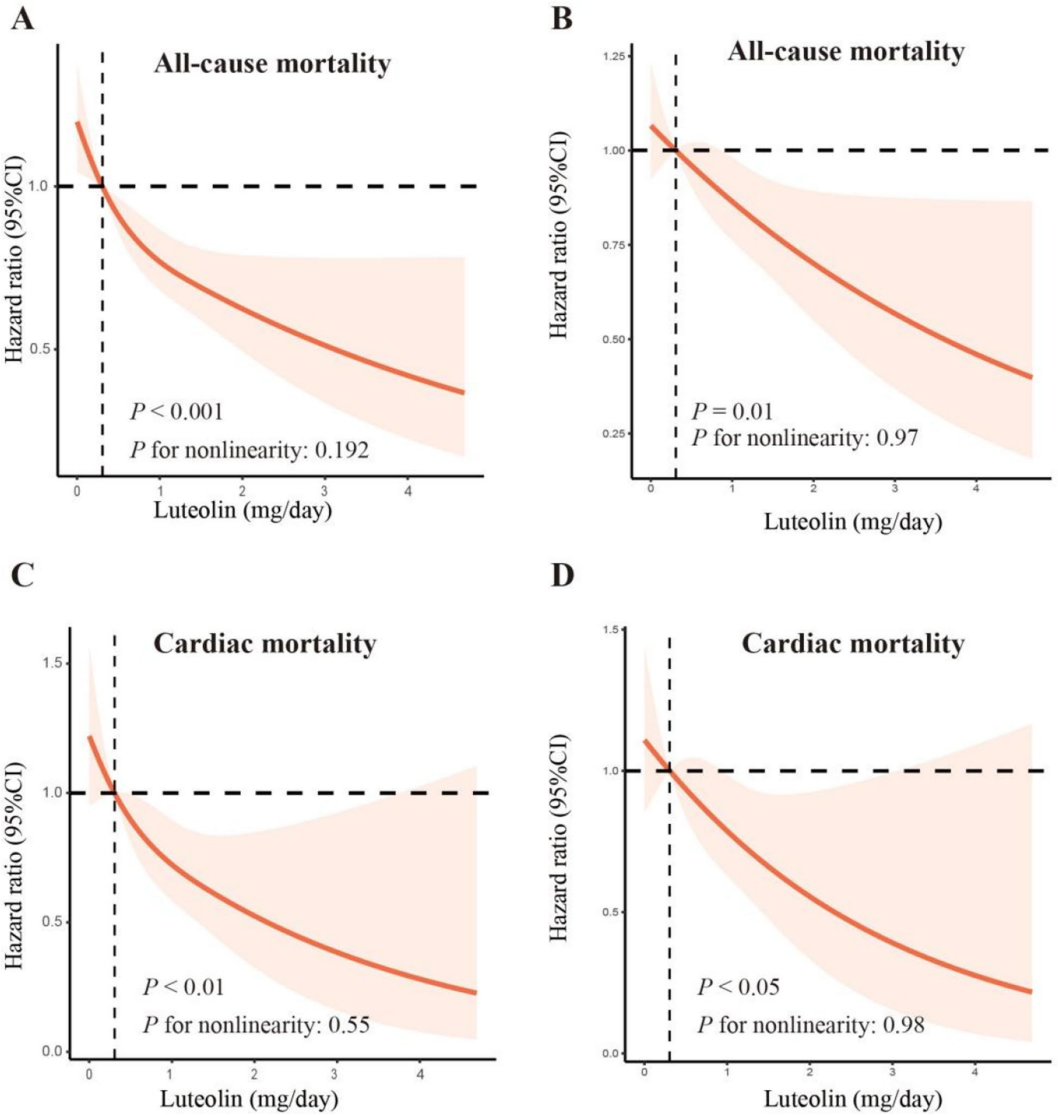
The dose–response association of Lut intake with all-cause mortality (**A**, **B**) and cardiac mortality (**C**, **D**) among CKD patients. The dose–response association of continuous Lut intake with mortality risk was visualized by the restricted cubic spline model. Three knots of the spline model were determined at specific distribution percentiles (33%, 66%, 99%). The y-axis represents the HR of a given Lut intake compared with the corresponding median. (All-cause mortality, unadjusted HR, A; adjusted HR, B; Cardiac mortality, unadjusted HR, C; adjusted HR, D.) The spline model was adjusted for consistent confounding factors, including age, gender, marital status, poverty, smoking, drinking, energy intake, hypertension, HbA1c, CVD history, serum creatinine, uACR(ln-transformed). The shadow area depicts the 95% confidence intervals

The median follow-up time was 93 months, and 682 all-cause deaths (217 CVD deaths) were recorded. According to the RCS analysis (Fig. 2A&C), there was an inverse dose–response association between Lut intake (range: 0–8.945 mg/day) with all-cause mortality (*p* < 0.05; *p* for non-linearity = 0.192) and cardiac mortality (*p* < 0.05; *p* for non-linearity = 0.55) without change points. The correlation remained after adjusting for covariates, including age, gender, marital status, poverty, smoking, drinking, energy intake, hypertension, HbA1c, CVD history, serum creatinine, and uACR, (Fig. 2B&D).

### Univariate and multivariate COX regression analysis

In the Cox regression analyses, the hazard ratios (HRs) with 95% confidence intervals (CIs) were calculated to assess the relationship between Lut intake tertiles and mortality outcomes. In the crude model, compared with tertile 1, the unadjusted-HRs for the tertiles 2 and 3 of Lut intake were 0.72 (95% CI: 0.54,0.94) and 0.59 (95% CI: 0.48,0.73) for all-cause mortality, and 0.72 (95% CI: 0.44,1.18) and 0.52 (95% CI: 0.34,0.81) for cardiac mortality, respectively (Table 2). Each unit increment of Lut intake (ln-transformed) was associated with a 27% reduction in all-cause mortality (unadjusted-HR = 0.73; 95% CI: 0.65,0.83; *P* < 0.001) and a 34% reduction in cardiac mortality (unadjusted-HR = 0.66; 95% CI: 0.48,0.91; *P* = 0.01) (Table 2). For all-cause mortality, In models 2, compared with tertile 1, the adjusted-HRs for the tertile2 and tertile3 of Lut intake were 0.81(95%CI:0.61,1.07), 0.71(95%CI: 0.53,0.94) respectively (Table 2). In models 3, compared with tertile 1, the adjusted-HRs for the tertile2 and tertile3 of Lut intake were 0.84(95%CI:0.64,1.11), 0.74(95%CI:0.54, 1.0) respectively (Table 2). Additionally, in all-cause mortality, each unit increment of Lut intake (ln-transformed) was associated with an 18% reduction in model 2 (0.82(95% CI:0.7,0.96)) and a 19% reduction in model 3 (0.81(95% CI:0.69,0.95)) (Table 2). For cardiac mortality, in model 1, compared with tertile 1, the adjusted-HRs for tertile 3 of Lut intake were 0.58(95%CI:0.34,0,98). There is a cardiac protective effect in Lut intake, but it did not reach statistical significance in other adjusted models.

**Table 2:** HR (95% CI) for all-cause and cardiac mortality based on Lut intake among CKD patients

Characteristic		Lut intake (mg/day)			Per-unit increment of Lut intake	p
		Tertile 1	Tertile 2	Tertile 3		
		[0–0.16)	[0.16–0.574)	[0.574–8.945]		
All-cause mortality						
	Crude model	ref	0.72(0.54,0.94)	0.59(0.48,0.73)	0.73(0.65,0.83)	< 0.0001
	Model 1	ref	0.78(0.59,1.03)	0.69(0.52,0.91)	0.82(0.70,0.96)	0.01
	Model 2	ref	0.81(0.61,1.07)	0.71(0.53,0.94)	0.82(0.70,0.96)	0.01
	Model 3	ref	0.84(0.64,1.11)	0.74(0.54,1.00)	0.81(0.69,0.95)	0.01
Cardiac mortality						
	Crude model	ref	0.72(0.44,1.18)	0.52(0.34,0.81)	0.66(0.48,0.91)	0.01
	Model 1	ref	0.77(0.44,1.33)	0.58(0.34,0.98)	0.71(0.49,1.04)	0.08
	Model 2	ref	0.82(0.47,1.44)	0.61(0.36,1.03)	0.72(0.50,1.02)	0.07
	Model 3	ref	0.85(0.48,1.50)	0.63(0.35,1.12)	0.71(0.49,1.02)	0.06

HR (95% CI) was estimated by Cox proportional hazards model and accounted for the sample weights. Cardiac mortality was defined as I00-I09, I11, I13, I20-I51 according to the ICD-10 criteria

Crude was unadjusted

model 1: age, gender, marital status, poverty, ethnicity, smoking, drinking, energy strata

model 2: age, gender, marital status, poverty, ethnicity, smoking, drinking, energy strata, hypertension, HbA1c strata, history of CVD

model 3: age, gender, marital status, poverty, ethnicity, smoking, drinking, energy strata, hypertension, HbA1c strata, history of CVD, creatinine, ACR(ln-transformed)

### Survival analysis

**Fig. 3:**
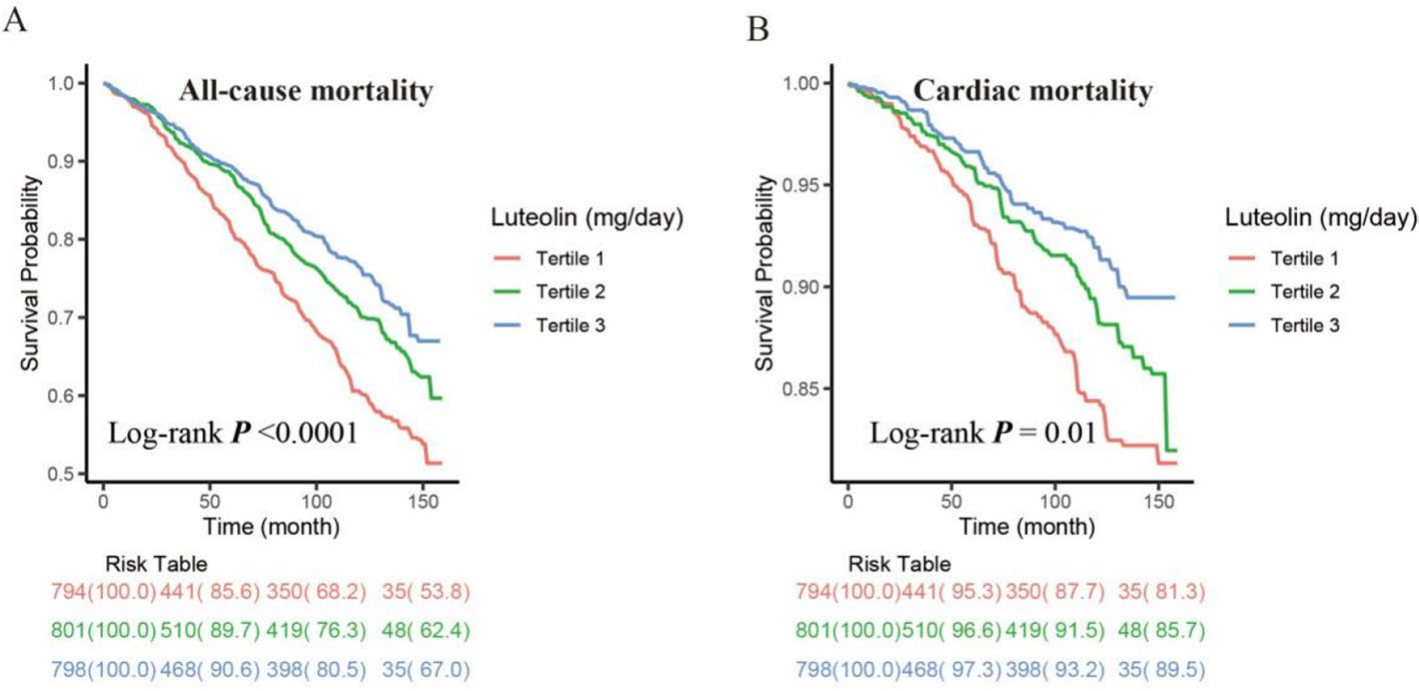
Kaplan–Meier survival curve of all-cause mortality (**A**) and cardiac mortality (**B**) based on Lut intake tertiles among CKD patients

The Kaplan–Meier survival analyses demonstrated that a higher intake of Lut was associated with a decreased risk of all-cause mortality (Log-rank *P* < 0.001) and CVD mortality (Log-rank *P* = 0.005) (Fig. 3).

### Sensitivity analysis

**Fig. 4:**
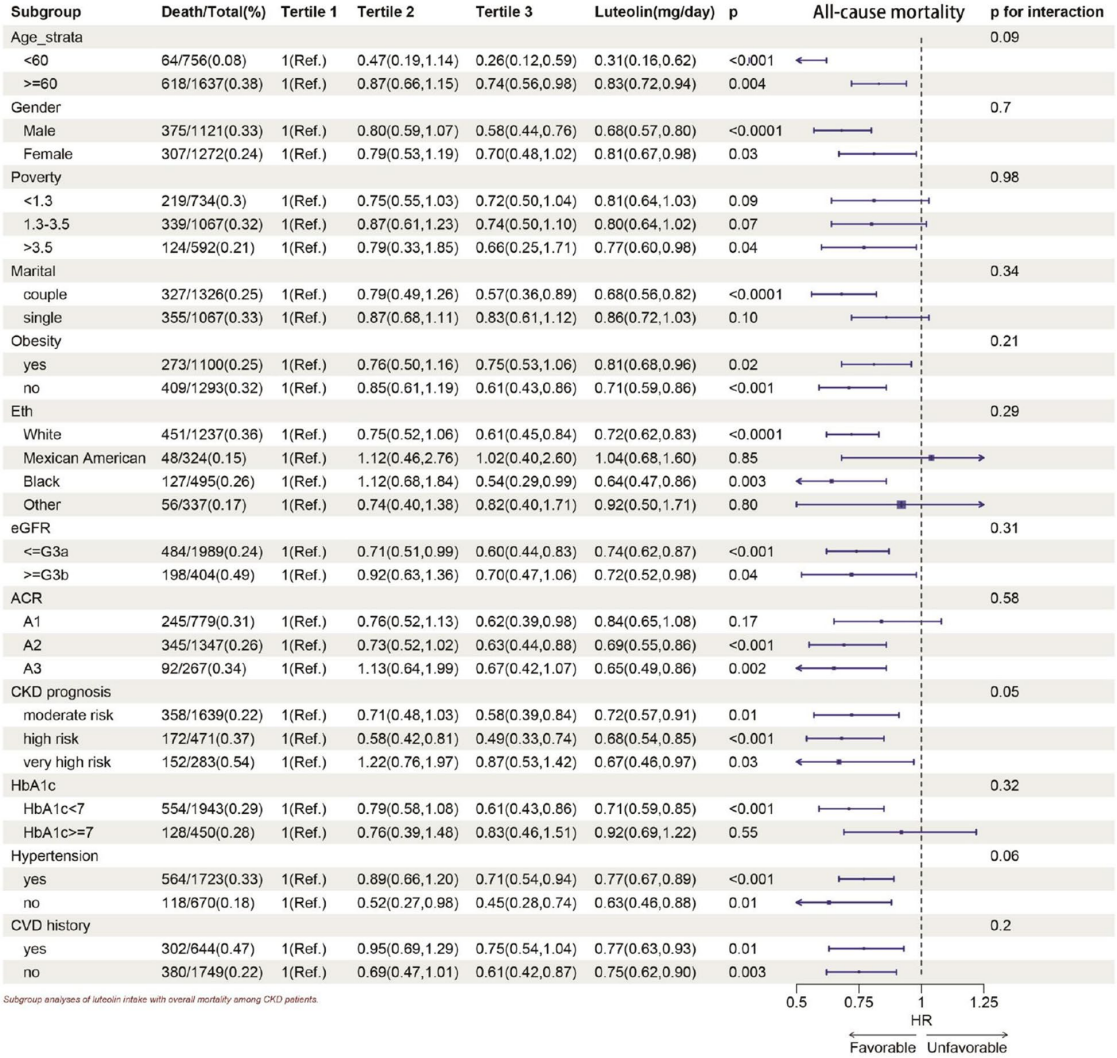
Subgroup analyses of Lut intake with all-cause mortality among CKD patients. HR (95% CI) was assessed by Cox proportional hazards model. The model was adjusted for covariates including age, serum creatinine, uACR(uACR Ln-transformed), BMI(weight/height squared), uric acid, blood urea nitrogen, bicarbonate, Hb, albumin, HbA1c. The interaction between Lut intake (continuous) and the stratified variable was assessed by the Wald test *P* < 0.05 was statistically significant

**Fig. 5:**
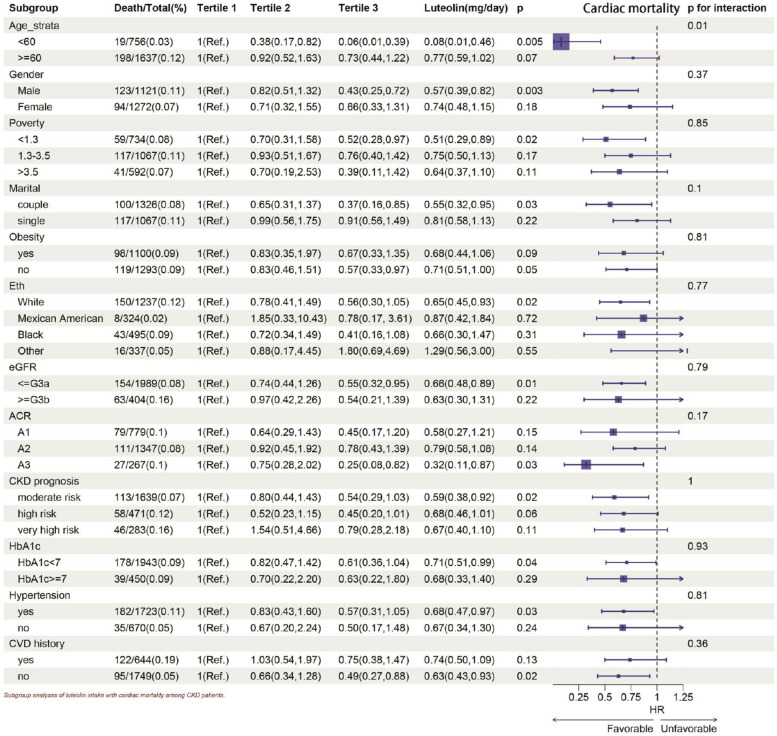
Subgroup analyses of Lut intake with cardiac mortality among CKD patients. HR (95% CI) was assessed by Cox proportional hazards model. The model was adjusted for covariates including age, serum creatinine, uACR(uACR Ln-transformed), BMI(weight/height squared), uric acid, blood urea nitrogen, bicarbonate, Hb, albumin, HbA1c. The interaction between Lut intake (continuous) and the stratified variable was assessed by the Wald test *P* < 0.05 was statistically significant

In subgroup analysis (Figs. 4 and 5), the Lut intake showed a negative correlation with all-cause mortality and CVD mortality. Significant associations were found between Lut intake and age, gender, ethnicity, marital status, obesity, white and black race, every stage of CKD, moderate and large urinary protein, CKD progression, Hb1Ac < 7, with and without hypertension, with and without CVD in relation to all-cause mortality. The inverse relationship between Lut intake and CVD mortality was especially noticeable in older participants, males, low income, white race, couples, non-obese, early CKD stage, moderate CKD progression risk, Hb1Ac < 7, hypertension, and no CVD history (Figs. 4 and 5).

## Discussion

The effective renal protective effect of Lut has been demonstrated by various animal studies. Fundamental studies in vivo using a mouse model have shown that levels of renal interstitial injury and fibrosis were significantly reduced when Lut was administered [17]. In angiotensin II (AngII) -induced apolipoprotein E-deficient (Apoe / -) mice, Liu et al. found that oral administration of Lut (100 mg / kg / day for 4 weeks) had a therapeutic effect on kidney injury, thus delaying the progression of kidney disease [18]. In MRL/lpr mice model, Lut can ameliorate pathological abnormalities and improve renal function by reducing renal oxidative stress and urinary protein levels, and can mitigate renal damage caused by infiltrating macrophages [19]. And Lut intake attenuates doxorubicin-induced derangements of the kidney by reducing oxidative and inflammatory stress to suppress apoptosis [20]. Another study showed that Lut in epimedium ameliorates IgA nephropathy and attenuates chronic renal failure [21]. This population-based study confirms for the first time that increasing dietary intake of Lut is negatively associated with all-cause and CVD mortality in CKD patients, and these are consistent with previous clinical and fundamental studies. Therefore, it is recommended that eating more Lut-rich foods may be a promising dietary intervention strategy for the treatment of CKD patients. Compared with drugs, dietary intervention is safer and more acceptable. At the same time, it can decrease the risk of cardiovascular death in patients with CKD. Therefore, increased intake of Lut in the diet may be an effective way to prevent CVD.

It is well-known that CKD patients have a markedly increased risk of CVD. In sensitivity analyses, except for cardiac mortality, higher Lut intake did not substantially correlate with major cause-specific mortality (diabetes, malignancy, and cerebrovascular disease). The decrease in cardiac mortality may be the main cause of improved prognosis for CKD. Lut has been proven to have cardio-protective effects both in vivo and in vitro in recent scientific research. The cardio protection was mainly derived from a reduction in cardiomyocyte apoptosis, a reduction in myocardial infarct size, and an increase in left ventricular ejection fraction [22]. First, in myocardial I/R injury, Lut enhances cardiomyocyte contraction by inhibiting apoptosis [23], blocking oxidative stress pathways [24], thereby attenuating I/R injury [25]. Second, in patients with heart failure, Lut improves cardiac function through modulation of cardiomyocyte contractility, enhancement of cardiomyocyte autophagy, and limitation of cardiac remodeling [26]. Furthermore, in atherosclerotic disease, Lut inhibits the vascular smooth muscle cell (VSMC) proliferation and migration, thereby reducing inflammation and ameliorating oxidative damage [27]. Moreover, there are epidemiological data suggesting that Lut intake reduces adult cardiac mortality in diabetic patients [28].

Although the exact mechanism behind the association between dietary Lut intake and decreased mortality risk in CKD patients remains unclear, several relevant hypotheses have recently been put forth. Research has demonstrated that Lut's anti-inflammatory, anti-apoptotic, and antioxidant properties may both prevent and attenuate renal failure [29]. Lut provides renal protection against several stimuli leading to renal injury, including sepsis, nephrotoxic drugs and renal ischemia [18, 30–32]. First, through antioxidant and free radical scavenging mechanisms, Lut reduces oxidative stress indicators and has renal protective properties [33, 34]. Second, Lut has an anti-inflammatory effect by reducing the expression of transcription factors and regulatory enzymes, as well as the synthesis of pro-inflammatory indicators and apoptosis-associated proteins [30, 35]. Furthermore, Lut suppresses the activity of the angiotensin-converting enzyme, which in turn suppresses the renin–angiotensin–aldosterone pathway. This reduces renal damage and delays the advancement of kidney disease [18]. Finally, Lut decreases the advancement of renal disease and improves the prognosis of renal function by reducing glomerulosclerosis and/or progressive interstitial fibrosis in obstructive nephropathy [36].

There are some findings that remain noteworthy in our study. The anti-inflammatory effects of Lut may be responsible for the improved cardiac-specific prognosis. Low-grade inflammation is a common feature in CKD and CVD [37]. The potent anti-inflammatory effect of Lut has been well established by previous studies. Inflammation plays a significant role in the progression, persistence, and worsening of CKD and a low-grade systemic inflammation is present as an underlying factor in CKD [38]. Consistently, our study indicated that CKD patients benefited from increasing daily Lut consumption in each stage of CKD. As CKD progresses, the benefits of Lut may become more pronounced. For each unit of increased Lut intake, there was a 26% reduction in early stage and 28% reduction in advanced CKD. The phenomenon may be due to Lut's anti-inflammatory effect. In addition, higher levels of inflammation mean a higher risk of CKD. Our study suggests that as the risk of CKD increases, the benefit of Lut intake progressively increases. With growing proteinuria, the higher the intake of Lut, the better the prognosis for CKD patients. For each unit of increased Lut intake, CKD patients with proteinuria over 300 mg/g had a 34% reduction in all-cause mortality reduction and a 68% reduction in cardiovascular mortality. Consistent with previous basic studies [18], Lut may improve pathological abnormalities by reducing renal oxidative stress and urinary protein levels, thereby improving prognosis.

Lut intake affects the prognosis of CKD patients, at the same time depending on patients with or without hypertension, altered blood glucose, and CVD. As is well known, CKD and diabetes are major causes of CVD mortality [39]. For CVD mortality, patients with HbA1c ≥ 7% had more reduction compared with patients with HbA1c < 7%. However, this reduction was not significant. In addition, a low-grade inflammatory state is a chronic process that gradually promotes heart disease progression [40]. Thus, the benefits of Lut intake may become more pronounced in CVD patients. In response to hypertension, glomerular microinflammation is initiated after activation of endothelial cells [3]. Consistently, for each unit of increased Lut intake, CKD patients with hypertension had a 23% reduction in all-cause mortality reduction and 32% reduction in CVD mortality. As a result, higher daily intake of Lut can significantly improve the prognosis of CKD patients with hypertension.

The strengths of our research include the use of a large sample size that represents adults across the United States, making it inclusive of the entire national population. This research is the initial exploration into the connection between dietary Lut intake and CVD and all-cause mortality in CKD. Furthermore, RCS was employed to investigate the correlation between Lut intake and CKD mortality, revealing a negative and linear correlation. Nevertheless, our research does have some constraints. It is important to mention that there were minor differences in the baseline characteristics of participants because some information on Lut factors was missing. The possibility of selection bias in the study could arise from this inconsistency. Furthermore, the use of self-administered surveys to assess health behavior metrics could potentially lead to misclassification bias. Moreover, the utilization of the 24-h recall method may introduce the possibility of recording bias, wherein participants may inadvertently overlook particular food items they consumed, resulting in an underestimation or overestimation of portion sizes. In addition, Lut widely exists in healthy food, distinguishing Lut intake from a healthy diet (especially one that contains other flavonoids) remains difficult, although potential dietary confounding factors (energy intake) have been adjusted in analyses. In the end, even though we tried to consider different possible factors that could affect the results, the limitations of an observational study design mean that there might still be some remaining confounding that cannot be completely removed. It must be stressed that as our findings are observational, it is important to be cautious before concluding that our findings demonstrate a causal relationship.

## Conclusion

Among a group of US adults that represents the entire nation, there was a significant correlation between higher Lut intake and a reduced CVD and all-cause mortality. This relationship was found to be linear and independent. Moreover, the correlation still exists within subgroups. The findings of our study suggest that daily Lut intake could potentially be beneficial in CKD patients. Further research is needed to investigate the relationship between daily Lut intake and CKD patients' mortality in a diverse population over a long period of time.

## Data Availability

The dataset for the National Health and Nutrition Examination Survey (NHANES) can be accessed by the public through the National Center for Health Statistics, which is a division of the Center for Disease Control and Prevention (CDC). The dataset can be found at the following website: https://www.cdc.gov/nchs/nhanes/.

## Abbreviations

Lut: Luteolin
CKD: Chronic kidney disease
I/R: Ischemia/reperfusion
NHANES: National Health and Nutrition Examination Survey
RCS: Restricted cubic splines
NCHS: National Center for Health Statistics
uACR: Urine albumin-to-creatinine ratio
eGFR: Estimated glomerular filtration rate
CKD-EPI: Chronic Kidney Disease Epidemiology Collaboration
MEC: Mobile Inspection Center
USDA: United States Department of Agriculture
FNDDS: Food and Nutrient Database for Dietary Studies
BMI: Body mass index
BP: Blood pressure
NDI: National Death Index
ICD: International Classification of Diseases
CVD: Cardiovascular disease
Hb: Hemoglobin
HRs: Hazard ratios
CIs: Confidence intervals
AngII: Angiotensin II
Apoe/: Apolipoprotein E-deficient
VSMC: Vascular smooth muscle cell
CDC: Center for Disease Control and Prevention
SBP: Systolic blood pressure
DBP: Diastolic blood pressure
